# Double Stimuli-Responsive di- and Triblock Copolymers of Poly(N-isopropylacrylamide) and Poly(1-vinylimidazole): Synthesis and Self-Assembly

**DOI:** 10.3390/ijms24010879

**Published:** 2023-01-03

**Authors:** Elena Yu. Kozhunova, Anna V. Plutalova, Andrey V. Sybachin, Alexander V. Chertovich, Elena V. Chernikova

**Affiliations:** 1 Faculty of Physics, Lomonosov Moscow State University, Leninskie Gory, 1, bld. 2, 119991 Moscow, Russia; 2 Faculty of Chemistry, Lomonosov Moscow State University, Leninskie Gory, 1, bld. 3, 119991 Moscow, Russia; 3 Semenov Federal Research Center for Chemical Physics, Kosygina, 4, 119991 Moscow, Russia

**Keywords:** reversible addition-fragmentation chain transfer (RAFT) polymerization, stimuli-responsive polymers, block copolymers, copolymers, self-assembly

## Abstract

For the first time, double stimuli-responsive properties of poly(N-isopropylacrylamide) (PNIPA) and poly(1-vinylimidazole) (PVIM) block copolymers in aqueous solutions were studied. The synthesis of PNIPA_60_-*b*-PVIM_90_ and PNIPA_28_-*b*-PVIM_62_-*b*-PNIPA_29_ was performed using reversible addition–fragmentation chain transfer (RAFT) polymerization. The polymers were characterized by size exclusion chromatography and ^1^H NMR spectroscopy. The conformational behavior of the polymers was studied using dynamic light scattering (DLS) and fluorescence spectroscopy (FS). It was found that PNIPA and block copolymers conformation and ability for self-assembly in aqueous medium below and above cloud point temperature depend on the locus of hydrophobic groups derived from the RAFT agent within the chain. Additionally, the length of PVIM block, its locus in the chain and charge perform an important role in the stabilization of macromolecular micelles and aggregates below and above cloud point temperature. At 25 °C the average hydrodynamic radius (R_h_) of the block copolymer particles at pH 3 is lower than at pH 9 implying the self-assembling of macromolecules in the latter case. Cloud points of PNIPA_60_-*b*-PVIM_90_ are ~43 °C and ~37 °C at a pH of 3 and 9 and of PNIPA_28_-*b*-PVIM_62_-*b*-PNIPA_29_ they are ~35 °C and 31 °C at a pH of 3 and 9. Around cloud point independently of pH, the *R_h_* value for triblock copolymer rises sharply, achieves the maximum value, then falls and reaches the constant value, while for diblock copolymer, it steadily grows after reaching cloud point. The information about polarity of microenvironment around polymer obtained by FS accords with DLS data.

## 1. Introduction

Stimuli-responsive, or smart, polymers attract growing interest due to multiple applications in various fields, including bioengineering [1,2], optics [3], electronics [4], etc. Among them, thermo- and pH-sensitive polymers cause special attention. These polymers are typically water-soluble, and their properties can be tuned readily by slight changes in the temperature or pH [5,6].

Poly(N-isopropylacrylamide) (PNIPA) is widely known as a temperature-responsive polymer [7,8]. PNIPA contains hydrophilic amido group and hydrophobic isopropyl group in its repeating unit that provide LCST phase behavior of PNIPA aqueous solutions [9]. LCST of PNIPA is about 32 °C [10], which is close to physiological temperature and is easy to adjust. Thus, PNIPA-based polymers have been developed for various biomedical applications, e.g., drug delivery systems [11,12], cardiovascular repair [13], sensing analysis [14], medical imaging [15], etc.

The shift of a hydrophilic–hydrophobic balance in PNIPA structure can be easily achieved by copolymerization of N-isopropylacrylamide with hydrophilic or hydrophobic monomers resulting in a shift of LCST to higher or lower values [16,17,18,19,20,21]. The incorporation of a hydrophilic unit causes the rise of LCST, while the introduction of hydrophobic unit results in the fall of LCST [22,23]. The similar approach can be used for any hydrophilic polymer [24,25,26].

In case N-isopropylacrylamide is copolymerized with ionizable functional vinyl monomers (e.g., containing a weak acid or a weak base group), the copolymer gets both temperature and pH-sensitive properties [27,28]. Positively charged random or block copolymers of N-isopropylacrylamide and amine-containing monomers, such as *N*,*N*-[(dimethylamino)propyl]methacrylamide [29] or ethylene imine [30,31], were synthesized, and investigated as carrier for gene delivery, and other variations [32,33].

Poly(1-vinylimidazole) belongs among weak bases; it can exhibit pH-sensitivity in aqueous solutions [34]. Poly(vinylimidazole)s, which contain heterocyclic aromatic rings on the polymer side chains, are of increasing interest for a wide variety of applications ranging from catalysis and polymeric ionic liquids to membrane material [35]. The stimuli-responsive properties of random and block copolymers of N-isopropylacrylamide and 1-vinylimidazole (VIM) are described in [36,37,38,39,40,41]. 

Block copolymers usually have more complex behavior than random copolymers. Combination of thermo-sensitive block with pH-sensitive block may lead to interesting properties. For example, diblock copolymer of PNIPA and poly(1-(4-vinylbenzyl)-3-methylimidazolium tetrafluoroborate) (PVBMI) exhibits an abnormal thermoresponsive phase transition at a temperature above the phase transition temperature of the PNIPA block [42]. Thermoresponsive behavior of diblock copolymers of poly(N-vinylcaprolactam) and PVIM depends on the length of the poly(N-vinylcaprolactam) block and the composition of aqueous solvent [43]. 

The stimuli-responsive properties of linear and star diblock copolymers of PNIPA and PVIM are discussed in [44,45]. Two diblock copolymers PNIPA*-b-*PVIM with a similar PNIPA block and different length of PVIM block were prepared by (reversible addition-fragmentation chain transfer) RAFT/MADIX polymerization [44]. The phase transition temperature is 2–4 °C higher for block copolymers, compared to PNIPA. The increase in the length of PVIM block gives rise to the growth of the temperature. The micellization of PNIPA-*b*-PVIM can be realized either by increasing temperature above the phase transition temperature of PNIPA block or by changing the solvent composition of methanol/water mixture (pH 7.4) at room temperature. In both cases, hydrophobic PNIPA-core micelles stabilized with PVIM corona are formed.

In the case of four-armed stars of poly(1-ethyl-3-vinylimidazolium bromide)-*b*-PNIPA, PNIPA formed either outer or inner segments [45]. The thermally induced phase separation behavior and assembled structure of the two series of star block copolymers are quite different, and depend on the sequence of each block, i.e., the locus of PNIPA along the arm of the star. The star block copolymer with block arms consisting of outer PNIPA block segments shows a two-step transition process. In contrast, the star block copolymer with inner PNIPA segments connected to the core exhibits a rapid aggregation.

Thus, the stimuli-responsive behavior of PNIPA and PVIM-based block copolymers depends on their composition and architecture. However, in all the mentioned studies dual stimuli-sensitivity of block copolymers has not been discussed. The authors limited their study to neutral pH (7.0–7.4). More interesting is the comparison of thermo-responsive properties in acidic and basic conditions, i.e., when VIM units are positively charged or uncharged (or weakly charged). Therefore, the aim of the present research is to perform the controlled synthesis of PNIPA and PVIM-based block copolymers using RAFT technique with terminal (diblock copolymer) and internal (triblock copolymer) locus of PVIM block and to study the effect of both temperature and pH on its conformational behavior in aqueous solutions.

## 2. Results and Discussion

### 2.1. Polymer Synthesis

**Scheme 1:**
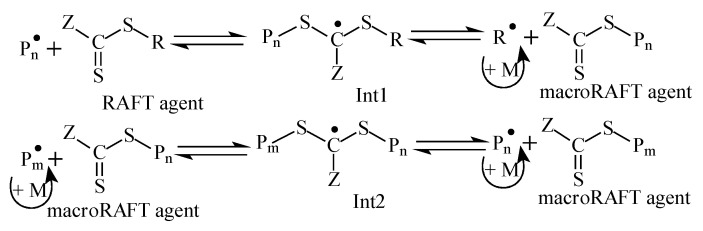
The general scenario of the RAFT mechanism.

The synthesis of block copolymers of vinyl monomers can be implemented by living anionic or various techniques of reversible deactivation radical polymerization (RDRP) [46]. However, a direct synthesis of PNIPA with narrow molecular weight distribution (MWD) and desired molecular weight (MW) is possible only in conditions of radical polymerization. Among other RDRP methods, RAFT polymerization is a versatile technique for the controlled synthesis of macromolecules with pre-determined architecture. It is based on the use of the special chain transfer agents of the general structure Z–C(=S)–S–R, where Z is a stabilizing group and R is a leaving group. The role of Z group is to stabilize the formation of radical intermediate (Int1), while R group provides the fragmentation of intermediate and further reinitiation of the polymerization (Scheme 1). 

When the Z and R groups are chosen correctly, the initial RAFT agent is rapidly transferred to the macroRAFT agent. If the latter participates actively in addition–fragmentation reaction with the propagating radical P_m_^•^, the formation of the polymer Z–C(=S)–S–P_m_ with narrow MWD and controlled MW occurs. When Z = –S–R, both R groups become leaving and the forming macroRAFT agent has a structure P_n_–S–C(=S)–S–P_n_.

**Scheme 2:**
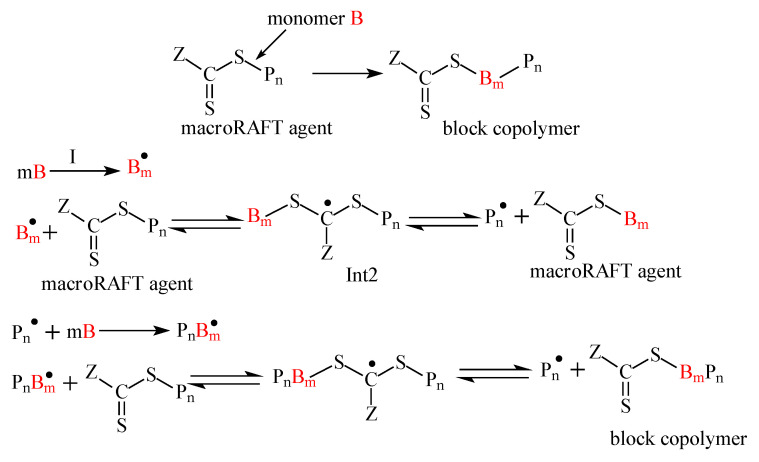
The general scenario of the formation of block copolymer.

In the case when the macroRAFT agent is added to the mixture of the monomer of another chemical nature and the initiator, the block copolymer is formed (Scheme 2).

**Scheme 3:**
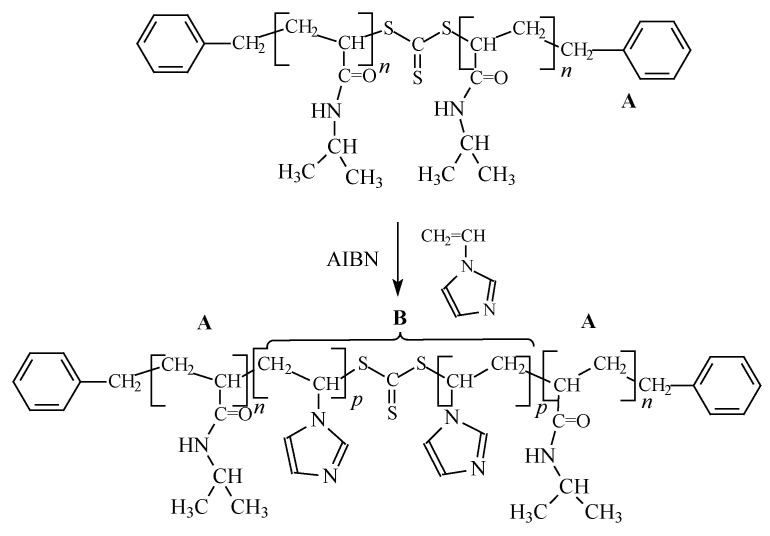
Formation of a triblock copolymer ABA.

RAFT polymerization is a preferred method of controlled synthesis of N-containing polymers among other techniques of RDRP [47]. Aiming to synthesize di- and triblock copolymers of PNIPA and PVIM, two various trithiocarbonates were used. Being symmetrical bifunctional RAFT agent (R–S–C(=S)–S–R, where R = –CH_2_Ph), dibenzyl trithiocarbonate (BTC) provides the formation of the macroRAFT agent (A) in polymerization of NIPA, i.e., the polymer with trithiocarbonate moiety within polymer chain [47,48]. Both polymeric substituents retain the properties of leaving groups. Thus, being used in chain extension with VIM, macroRAFT-agent A forms symmetrical triblock copolymer ABA PNIPA-*b*-PVIM-*b*-PNIPA (Scheme 3).

**Scheme 4:**
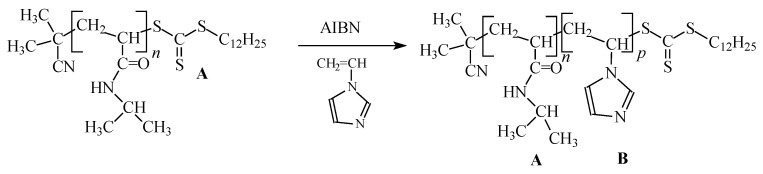
Formation of a diblock copolymer AB using monofunctional RAFT agent.

The synthesis of diblock copolymer AB PNIPA-*b*-PVIM is performed using monofunctional RAFT agent [47], e.g., 2-cyano-2-propyl dodecyl trithiocarbonate (CPDTC) (Scheme 4).

**Figure 1:**
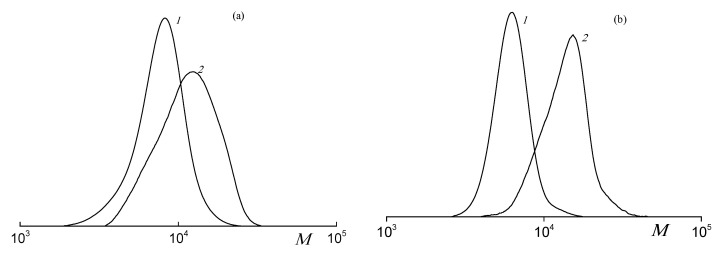
SEC traces of PNIPA (1), and block-copolymers (2), synthesized with BTC (**a**), and CPDTC (**b**).

Thus, on the first step, two macroRAFT agents were synthesized using bifunctional BTC (PNIPA-1) and monofunctional CPDTC (PNIPA-2). The formulations of reaction mixtures for macro-RAFT agent synthesis are listed in the Section 3. The 70-fold molar excess of RAFT agent with respect to AIBN provides the formation of the living chains. According to size exclusion chromatography (SEC), PNIPA-1 has M_n_ = 7.1 kg⋅mol^−1^ and Đ = 1.13 and PNIPA-2 has M_n_ = 6.0 kg⋅mol^−1^ and Đ = 1.07 (Figure 1, curves 1).

**Figure 2:**
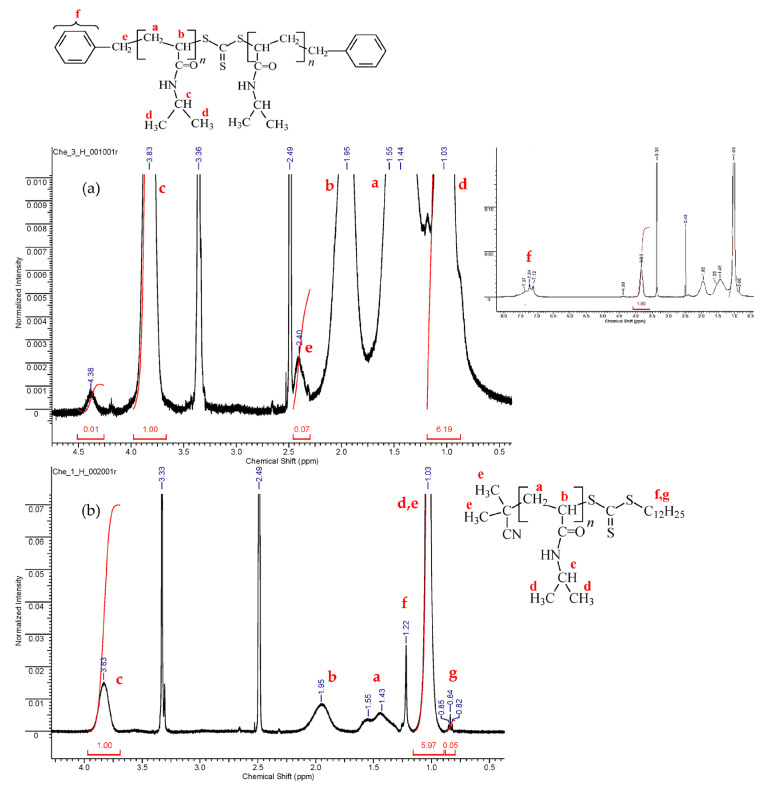
^1^H NMR spectra of PNIPA-1 (**a**) and PNIPA-2 (**b**) in DMSO-d_6_.

For direct determination of M_n_, ^1^H NMR spectroscopy was used. ^1^H NMR spectra of PNIPA-1 and PNIPA-2 in DMSO-d_6_ are presented in Figure 2. In both spectra, the characteristic peaks of PNIPA are observed. 

The resonance of protons of –NH–**CH**(CH_3_)_2_ is observed at 3.83 ppm, protons of –NH–CH(**CH_3_**)_2_ at 1.03 ppm, **CH_2_** and **CH** protons of backbone at 1.43–1.55 and 1.95 ppm, respectively. The structure of PNIPA-1 and PNIPA-2 differs in the chemical nature of the terminal groups: C_6_H_5_CH_2_– for PNIPA-1 and (CH_3_)_2_(CN)C– and (CH_2_)_11_CH_3_ for PNIPA-2. In the spectrum of PNIPA-1 the resonance of protons of C_6_H_5_CH_2_ group connected with CH_2_ group of backbone is observed at 2.40 ppm (Figure 2a). The residual BTC manifests itself in the signal observed at 4.38 referred to the protons of C_6_H_5_CH_2_ group connected with sulfur atom. Thus, the signal at 7.0–7.5 ppm corresponding to aromatic protons cannot be used for calculation of polymerization degree P_n_. To estimate P_n_, the integral intensities of protons of isopropyl group –NH–**CH**(CH_3_)_2_ (3.83 ppm, *I*_CH_) and C_6_H_5_CH_2_ group (2.40 ppm, *I*_CH2_) were used. Considering that macromolecule contains two benzyl groups, it is easy to estimate P_n_ as:

$$\frac{I_{\mathrm{CH}}/1}{I_{\mathrm{CH}2}/4}=\left(1/1\right)/\left(0.07/4\right)=57$$

In the spectrum of PNIPA-2 the signal of protons of (CH_3_)_2_(CN)C– is overlapped with methyl protons of isopropyl group of PNIPA (1.03 ppm), while methylene protons of C_12_H_25_ group are observed at 1.22 ppm [49]. The signal of methyl protons of C_12_H_25_ group is observed at 0.82–0.85 ppm (Figure 2b). Thus, to estimate P_n_, the protons of isopropyl group –NH–**CH**(CH_3_)_2_ (3.83 ppm) and CH_3_ group (0.82–0.85 ppm) were used. Considering that macromolecule contains two benzyl groups, it is easy to estimate P_n_ as:

$$\frac{I_{\mathrm{CH}}/1}{I_{\mathrm{CH}3}/3}=\left(1/1\right)/\left(0.05/3\right)=60$$

Thus, both macro-RAFT agents have the similar MW.

**Figure 3:**
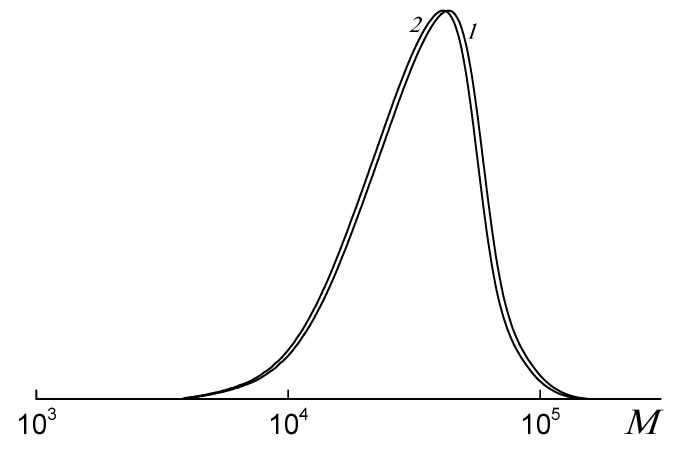
SEC traces of PNIPA before (1) and after (2) heating at 70 °C in glacial acetic acid.

In the second step, both macroRAFT agents were programmed to use in the polymerization of VIM. However, RAFT polymerization of VIM in conventional organic solvents and in aqueous media suffers from side reactions resulting in the formation of oligomer with low yield [43]. Recently, it was shown that well-controlled and low dispersity (Đ = 1.05) PVIM could be synthesized via RAFT polymerization by using acetic acid as a special solvent [50]. Similarly, controlled radical polymerization of 4-vinylimidazole was achieved in glacial acetic acid mediated with 4-cyano-4-(ethylsulfanylthiocarbonylsulfanyl)pentanoic acid [49]. Thus, the synthesis of block copolymer was suggested to perform in acetic acid. We have proved initially that PNIPA would not be subjected to acidic hydrolysis in polymerization conditions. Aiming this, PNIPA was dissolved in glacial acetic acid. The prepared solution was poured into an ampoule, degassed; the ampoule was sealed, and put into thermostat preheated at 70 °C for 72 h. After that, the solution was subjected to dialysis against distilled water and lyophilized. If hydrolysis occurs, then amide group is converted to carboxylic one. However, the aqueous solutions of PNIPA before and after hydrolysis were characterized by pH 7. The spectra obtained by Fourier-transform infrared spectroscopy of both polymers are similar; the same characteristic bands of PNIPA are observed: 3400–3300 cm^–1^ referred to NH group, 2970–2850, 1370–1450, and 1180–1100 cm^–1^ belongs to CH, CH_2_, and CH_3_ groups; 1646 cm^–1^ referred to Amide I and 1546 cm^–1^ to Amide II [51]. SEC traces of the polymers are in complete record (Figure 3). Thus, it may be concluded, that no changes in chemical structures occur upon heating of PNIPA in acetic acid in inert atmosphere.

**Figure 4:**
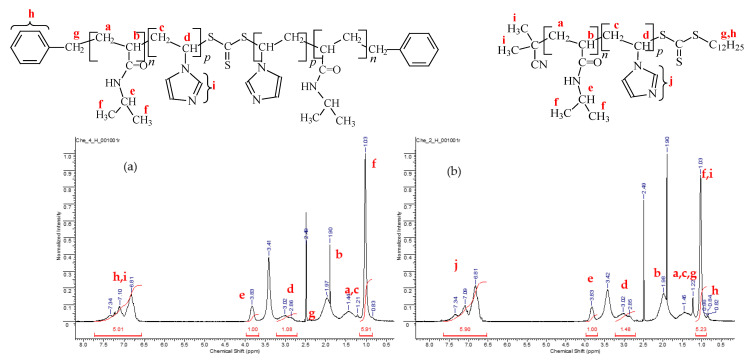
^1^H NMR spectra of block copolymers synthesized using PNIPA-1 (**a**) and PNIPA-2 (**b**) in DMSO-d_6_.

The chain extension of PNIPA-1 and PNIPA-2 with VIM was performed in similar conditions. SEC traces of corresponding block copolymers are given in Figure 1. Both block copolymers are characterized by unimodal narrow MWD, thus all of the initial macroRAFT agent was consumed. The block copolymers were analyzed by ^1^H NMR spectroscopy (Figure 4). Along with the signals of PNIPA protons, the signals corresponding to the VIM protons appear in the spectrum. The signals at 6.8–7.3 ppm are referred to protons of imidazole ring, the signal at 2.8–3.0 ppm is referred to CH protons of PVIM backbone. The signal at 3.41 ppm is due to the bound water [49]. To estimate the block copolymer composition, the protons of isopropyl group –NH–**CH**(CH_3_)_2_ (3.83 ppm) and CH protons (2.8–3.0 ppm) of PVIM backbone were used:

$$\frac{F_{\mathrm{NIPAM}}}{F_{\mathrm{VIM}}}=\frac{I_{\mathrm{NIPAM}}/\left(I_{\mathrm{NIPAM}}+I_{\mathrm{VIM}}\right)}{I_{\mathrm{VIM}}/\left(I_{\mathrm{NIPAM}}+I_{\mathrm{VIM}}\right)}$$

where *F*_NIPAM_ and *F*_VIM_ are the molar parts of NIPAM and VIM units in the copolymer, *I*_NIPAM_ and *I*_VIM_ are the integral intensities of the signals corresponding to the protons of isopropyl group –NH–**CH**(CH_3_)_2_ at 3.83 ppm, and CH protons of PVIM backbone at 2.8–3.0 ppm.

Thus, the block copolymer synthesized from PNIPA-1 contains 48 mol. % of NIPA and 52 mol. % of VIM, respectively, while synthesized from PNIPA-2–40 mol.% of NIPA and 60 mol.% of VIM, respectively. Knowing P_n_ of PNIPA and block copolymer composition, P_n_ of PVIM block may be calculated and as a consequence, MW of block copolymer. The results of calculation of polymerization degree of PVIM block and M_n_ of the block copolymers are given in Table 1. Hereafter, the block copolymer synthesized using PNIPA-1 is specified as PNIPA_28_-*b*-PVIM_62_-*b*-PNIPA_29_, and block copolymer synthesized using PNIPA-1 is specified as PNIPA_60_-*b*-PVIM_90_.

### 2.2. DLS Study of the pH- and Temperature-Dependent Self-Organization of Block-Copolymers

**Figure 5:**
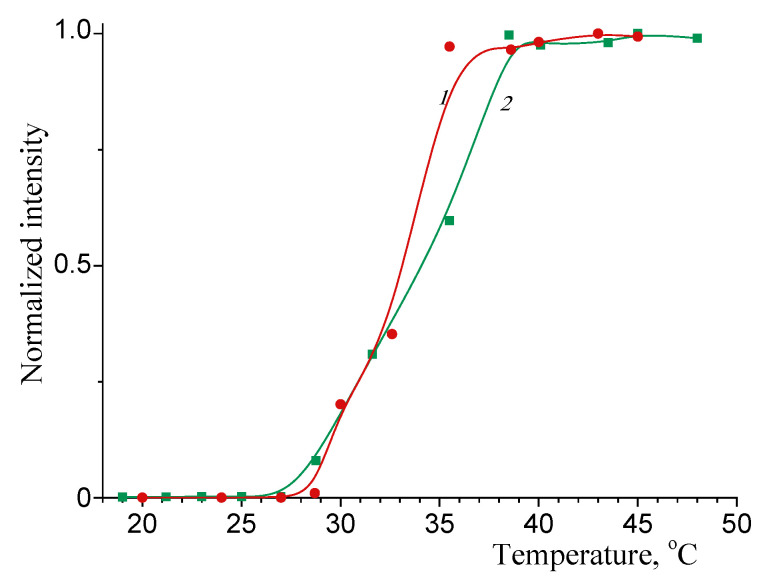
The dependence of normalized scattered light intensity on temperature of PNIPA-1 (1) and PNIPA-2 (2) aqueous solutions measured at the angle of 90° pH = 7.0.

**Figure 6:**
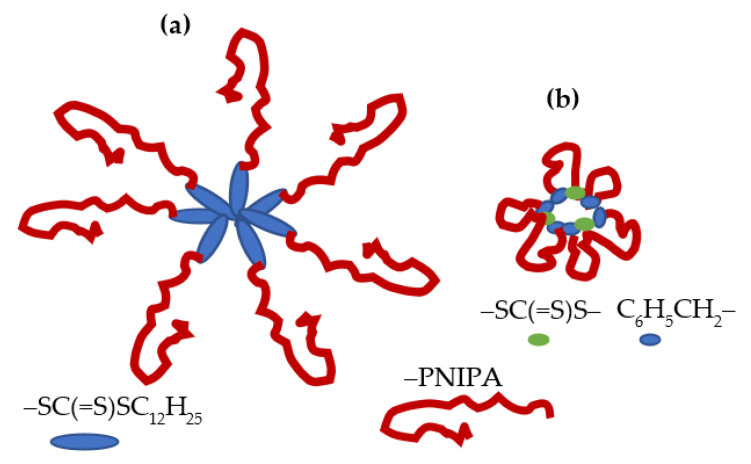
The proposed micelle structures of PNIPA-2 (**a**) and PNIPA-1 (**b**) in aqueous solutions before cloud point temperature.

In aqueous medium, PNIPA demonstrates temperature-responsive properties. It is known to collapse in water at temperatures 32 °C and above, representing LCST behavior. Cloud point temperatures (T_cp_) of PNIPA macroRAFT were determined from the temperature dependences of light scattering as the half-width of a sharp increase in the normalized scattered light intensity determined by dynamic light scattering (DLS) method (Figure 5 and Table 2). PNIPA-based macroRAFT agents show thermo-responsive behavior typical for PNIPA polymer with the cloud point temperature about 32 °C independently of pH of their aqueous solutions. Although PNIPA-1 and PNIPA-2 differ slightly in the values of the polymerization degree (57 and 60, respectively), the transition from transparent to turbid solution occurs at the different temperature range ΔT_cp_ (Figure 5, Table 2). Additionally, the difference in the values of the average hydrodynamic radii (R_h_) of both PNIPA samples before cloud point is noticeable (Table 2). Rough estimate of the mean square distance between the chain ends <h^2^>^1/2^ = n^1/2^l (n—number of monomer units and l = 0.25 nm—the length of monomer unit) gives the value of 1.9 nm, which allow the evaluation of the radius of gyration <R_g_^2^> = <h^2^>/6~0.6 nm^2^ or <R_g_^2^>^1/2^~0.8 nm. Thus, for PNIPA-1 the observed value of R_h_ is close to estimated value of <R_g_^2^>^1/2^, while for PNIPA-2 it exceeds <R_g_^2^>^1/2^ in ~10 times. Thus, it may be concluded that the conformational behavior of both PNIPA samples in aqueous solutions below cloud point is different. We may suppose that PNIPA-2, containing hydrophilic NIPA units and bulky hydrophobic C_12_H_25_ terminal group, is able to form micelles-like surfactants with hydrophobic core and hydrophilic corona (Figure 6a). While PNIPA-1 with smaller benzyl terminal groups exists in the form of single chain coils or small flower-like aggregates [52] (Figure 6b). Upon heating, PNIPA-1 loses its solubility and aggregates in relatively narrow temperature range. While PNIPA-2 needs the additional rearrangement to undergo phase separation, which leads to widening of the temperature range required for this transition.

PVIM contains imidazole group that exhibits the properties of both a weak base (pK_b_ = 7.0) and a weak acid (pK_a_ = 14.9); thus, it reveals pH-responsive properties. Thus, in aqueous medium PNIPA and PVIM-based di- and triblock copolymers are expected to demonstrate both pH- and temperature-responsive properties inherited from the comprising species. At the same time, the respective position along the chain of the blocks with different sensitivity performs an important role in the possible self-assembled structures. To ascertain the influence of pH, temperature, and chain architecture on the behavior of the obtained block-polymers, we studied their aqueous solutions by DLS method.

The block copolymer samples of PNIPA_60_-*b*-PVIM_90_ and PNIPA_28_-*b*-PVIM_62_-*b*-PNIPA_29_ were dissolved in deionized water at two different pH values—3 and 9—at the weight polymer concentration of 1 mg/mL and were left to reach the equilibrium condition for two days. Basing on the equation:

pK_b_ = pOH − lg(α/(1 − α))

where pK_b_ is the basicity constant, pOH = 14 − pH, and α is the degree of ionization; one can easily estimate that VIM units are completely charged at pH 3 and practically uncharged at pH 9.

In all cases, the polymers dissolved in water without precipitate. Furthermore, with the help of ALV compact goniometer, the dependencies of the scattering intensity and hydrodynamic radius R_h_ on temperature were measured. The addition on PVIM block, its length, position in the polymer chain, and pH of the solution significantly change the hydrophilicity of the polymer and, as a consequence, its cloud point (Table 2). 

**Figure 7:**
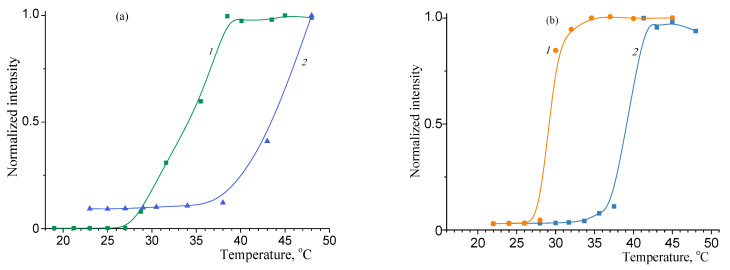
The dependence of normalized scattered light intensity on temperature of PNIPA_28_-*b*-PVIM_62_-*b*-PNIPA_29_ (1) and PNIPA_60_-*b*-PVIM_90_ (2) aqueous solutions measured at the angle of 90°. The pH = 3.0 (**a**) and 9.0 (**b**), respectively.

At pH 3, VIM units are charged resulting in the growth of the block copolymer hydrophilicity. In this case, the increase in the content of VIM units results in the shift of cloud point temperature to the higher values (Figure 7a). At pH 9, VIM units are uncharged and less hydrophilic; however, the PVIM block retains its solubility though it is lower than at pH 3. As a consequence, cloud point of block copolymer increases when VIM is ionized and decreases when it is uncharged. When polymerization degrees of PNIPA and PVIM blocks are close, the cloud point temperature of triblock copolymer is similar to pure PNIPAM. In the case of diblock copolymer that contains more VIM units, the solubility of the block copolymer increases rising cloud point temperature. Thus, the higher the VIM content in block copolymer, the higher the cloud point value (Figure 7b).

**Figure 8:**
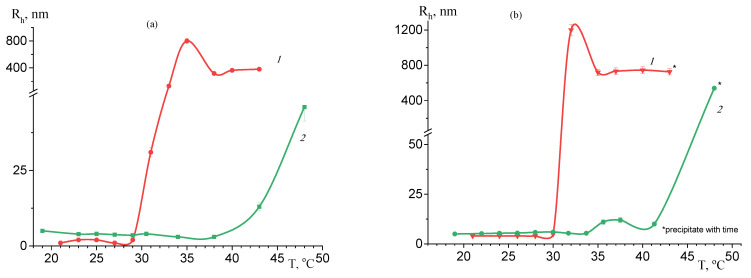
The temperature dependencies of hydrodynamic radius R_h_ at pH 3 (**a**) and pH 9 (**b**) for aqueous solutions of PNIPA_28_-*b*-PVIM_62_-*b*-PNIPA_29_ (1) and PNIPA_60_-*b*-PVIM_90_ (2); the measurements were taken under the scattering angle of 90°. * The sample precipitates with time when heated a few degrees above the Tcp.

The more interesting is temperature behavior of block copolymers around cloud point at various pH values (Figure 8). Below cloud point, R_h_ of PNIPA_28_-*b*-PVIM_62_-*b*-PNIPA_29_ at a pH of 3 and 9, respectively, is higher than for pure PNIPA-1 due to the presence of PVIM block. However, R_h_ of triblock copolymer at pH 3 is twice lower than at pH 9, although VIM units are charged at pH 3 and practically uncharged at pH 9 (Figure 8, curve 1, Table 2). It may be assumed that at pH 3 triblock copolymer exists in the form of single chain coils due to the charged middle block of PVIM, while at pH 9 it forms micelles with PVIM in the core and PNIPA in the corona. In the case of diblock copolymer PNIPA_60_-*b*-PVIM_90_, *R_h_* at pH 3 and 9 is smaller than for pure PNIPA-2, indicating that incorporation of PVIM block between NIPA units; C_12_H_25_ group prevents the micelle formation as it was in the case of PNIPA-2.

Near but below cloud point temperature, R_h,_ of all the block copolymers, decreases due to the loss of the solubility of PNIPA block. After that, aggregation takes place and R_h_ values increase sharply. However, the difference is observed for tri- and diblock copolymers. The R_h_ value of the former stabilizes indicating that rearrangement of aggregates may take place, while the R_h_ value of the later continues to grow. This difference may come from the various structure of block copolymers rather than different length of PVIM block.

Typical number-averaged distributions of hydrodynamic radius R_h_ observed at different temperatures and pH are shown in Figure S1 illustrating various behavior of block copolymers. 

To summarize, it is known that the polymers with hydrophobic RAFT agent groups tend to form a small number of bigger aggregates even in the conditions of good solvent [43] and effect on the intensity-averaged distributions. In general, the obtained block copolymers at the temperatures below T_cp_ have the characteristic R_h_ as 1–10 nm, which can be attributed to the single chain coils or small aggregates of a few macromolecules. When heated above T_cp_, all the species at both pH form large aggregates between 50 and 1000 nm, depending on the exact conditions, with the formation of quite monodisperse objects. It should be noted that both di- and triblock copolymers at pH 9 precipitate with time when heated several degrees above the critical temperature. At pH 3, none of precipitation is observed in the studied temperature range. Scanning electron microscopy images of the stable aggregates at pH 3 are presented in Figure S2. 

### 2.3. Fluorescence Spectroscopy Study of the pH- and Temperature-Dependent Behavior of PNIPA and Block-Copolymers

The ability of the polymers to incorporate hydrophobic molecules was studied using pyrene probe by a technique described elsewhere [53]. The π* → π emission spectrum of pyrene has five well-resolved vibronic bands at 370–400 nm. The first band undergoes significant intensity changes with variation of solvent polarity in contrast to the third band [54,55]. Thus, the ratio of the emission intensities of the first and the third bands is the measure of solvent polarity and is known as Py-scale [56]. The Py-scale has been widely used to characterize the polarity of structured or anisotropic media like micelles, biological membranes, or polymer blends [57,58]. Fluorescence technique can also give information about microenvironment around polymer segments and interactions between various groups in polymer systems [59]. The thorough analysis of PNIPA behavior below and above LCST using pyrene probe is described in [60,61].

**Figure 9:**
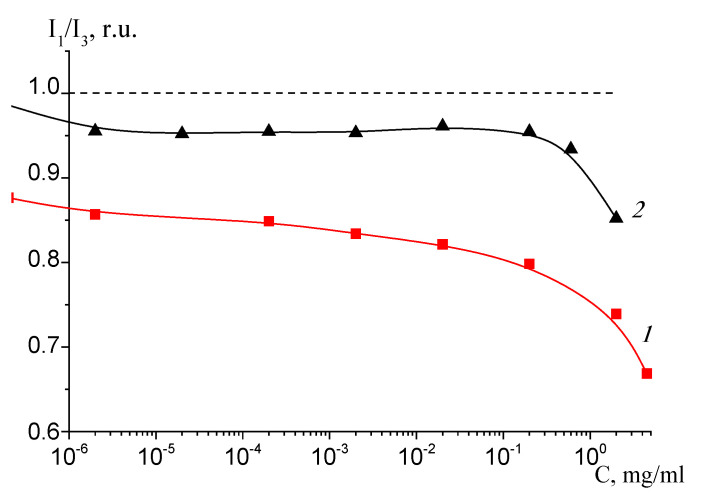
The dependence of peaks intensity ratio I_1_/I_3_ of the pyrene probe on the polymer concentration in acetate buffer at pH 4.5 at 25 °C for PNIPA-1 (1) and PNIPA-2 (2). Here and below, pyrene probe concentration 5 × 10^−7^ mol/L.

In the present research, experiments were performed in aqueous buffer solutions at pH 4.5 (acetate buffer) and pH 9 (Tris buffer) below (25° C) and above LCST (55° C). The fluorescence spectra of pyrene in the absence and in the presence of polymers are recorded and the ratio of intensities of the first and the third emission peaks I_1_/I_3_ is analyzed. In the absence of the polymer, the ratio of I_1_/I_3_ at pH 4.5 (acetate buffer) and at pH 9 (Tris buffer) were taken equal to 1.0 for each series of experiments. Figure 9 presents the normalized to buffer dependence of I_1_/I_3_ value of the polymer concentration for PNIPA-1 (curve 1) and PNIPA-2 (curve 2) at pH 4.5 at 25° C. 

In both cases, normalized I_1_/I_3_ value is lower than the value of the pure solution of the probe reflecting slight hydrophobic microenvironment of pyrene due to polymer backbone and the groups of RAFT agent incorporated in the chain. Moreover, PNIPA-2 is more hydrophilic than PNIPA-1. PNIPA-1 contains two hydrophobic benzyl groups at the chain ends and one hydrophobic trithiocarbonate group in the mid-chain, while PNIPA-2 contains polar (CH_3_)_2_(CN)C group on the α-end of the chain and hydrophobic alkyl group on its ω-end. No significant change in polarity of the probe is found in the wide range of the PNIPA concentrations up to 0.5 mg/mL. At higher PNIPA concentrations the decrease in I_1_/I_3_ value is observed reflecting increase in non-polar areas due to micelle formation or aggregation. These results work well with the DLS data (Table 2). In the case of PNIPA-2 the micelles with R_h_ of 8 nm may form with hydrophobic core of alkyl end-groups and hydrophilic corona of PNIPA. In the case of PNIPA-1 the flower-like small aggregates may form as it was discussed above. With the increase in the temperature up to 55 °C (~20 °C above LCST), the PNIPA chains collapse and the polarity of the microenvironment of the probe remains constant I_1_/I_3_ = 0.9–0.95. In the alkali medium, trithiocarbonate group is less stable; nevertheless, the similar results were observed both at 25 °C and 55° C, respectively, as at pH 4.5.

**Figure 10:**
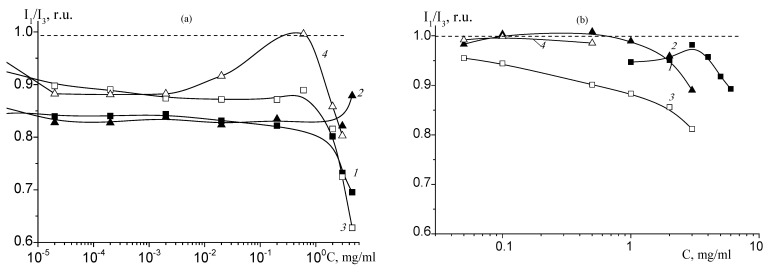
The dependence of peaks intensity ratio I_1_/I_3_ of the pyrene probe on the polymer concentration in acetate buffer at pH 4.5 (**a**) and pH 9 (**b**) at 25 °C (1, 2) and 55 °C (3, 4) for PNIPA_28_-*b*-PVIM_62_-*b*-PNIPA_29_ (1, 3) and PNIPA_60_-*b*-PVIM_90_ (2, 4). *Note*: no correct data was obtained for the samples PNIPA_60_-*b*-PVIM_90_ at concentrations above 0.5 mg/mL at 55 °C due to significant phase separation.

The behavior of block copolymers is more complex due to the presence of both thermoresponsive PNIPA and pH-responsive PVIM blocks and is sensitive to the locus of the block and the length of pH-responsible block. Figure 10 presents the dependence of peaks intensity ratio I_1_/I_3_ of the pyrene probe for block copolymers in acidic (a) and alkali (b) media.

At pH 4.5 VIM units in both block copolymers are charged. At 25 °C triblock-copolymer PNIPA_28_-*b*-PVIM_62_-*b*-PNIPA_29_ with internal PVIM block behaves itself similarly to PNIPA-1 except the decrease in I_1_/I_3_ value occurs at higher polymer concentration (Figure 10a, curve 1). At the same time for diblock copolymer PNIPA_60_-*b*-PVIM_90_ the pyrene probe retains its polarity in a wider range of polymer concentrations due to higher length of the charged PVIM block as compared to triblock copolymer (Figure 10a, curve 2). At 55 °C PNIPA block loses it solubility and collapses, while PVIM block retains its high solubility, resulting in the increase in the total polarity of the chain and I_1_/I_3_ values for block copolymers increase as compared to 25° C. As a whole, triblock copolymer behave itself similarly at both 25 °C and 55 °C (Figure 10a, curves 1, 3). Meanwhile, the polarity of the microenvironment increases with rise of the concentration of diblock copolymer containing longer PVIM block and slightly decreases with further increase in the polymer concentration (Figure 10a, curve 4). 

Increase in the pH to 9 results in partial deprotonation of the amino groups in PVIM block. Unexpectedly, the I_1_/I_3_ value is higher than in the case of acidic conditions (Figure 10b). The behavior of both block copolymers (Figure 10b, curves 1,2) at 25 °C resembles PNIPA polymers (Figure 9). However, the difference is observed upon heating at 55° C. Diblock copolymer precipitates after its concentration in solution exceeds 0.5 mg/mL (Figure 10b, curve 4), while triblock copolymer retains its solubility even at concentration of 3 mg/mL. At the same time, its hydrophobicity increases resulting in the decrease in I_1_/I_3_ value.

The results obtained by fluorescent method for both PNIPA and block copolymers accord with DLS results.

## 3. Materials and Methods

### 3.1. Materials and Polymer Synthesis

N-isopropylacrylamide (97%), RAFT agents dibenzyl trithiocarbonate (BTC, C_6_H_5_CH_2_–S–C(=S)–S–CH_2_C_6_H_5_, 97%) and 2-cyano-2-propyl dodecyl trithiocarbonate (CPDTC, (CH_3_)_2_(CN)C–S–C(=S)–S–C_12_H_25_, 97%), glacial acetic acid (100%) were used without further purification. Initiator 2,2′-azobisisobutyronitrile (AIBN, 97%) was recrystallized from ethanol and initiator potassium persulfate was used without further purification. Monomer 1-vinylimidazole (>99%), the solvents—1,4-dioxane and N, N-dimethyl formamide (DMF) were distilled before the use. Pyrene fluorescent probe was used without purification. All reagents were purchased from Sigma-Aldrich (St. Louis, MO, USA).

Two macro-RAFT agents were synthesized according to the following general procedure [62]. The given amount of NIPA was dissolved in 1,4-dioxane. This solution was added to the weighted amount of corresponding RAFT agent. Finally, the initiator was added as 1,4-dioxane solution. The final mixture was poured into ampoule. The reaction mixture was degassed through four freeze−pump−thaw cycles, and the ampoule was sealed. Next, it was immersed into the thermostat pre-heated at 80 °C for 48 h. After polymerization, the reaction mixture was cooled in liquid nitrogen, then diluted with 1,4-dioxane and dried by lyophilization in a vacuum. After that PNIPA was dissolved in water–1,4-dioxane mixture, then subjected to dialysis against water using dialysis sac with cut-off 3 kDa for 3 days and lyophilized. The monomer conversion was determined by gravimetry. Table 3 summarizes the formulations of reaction mixtures for macro-RAFT agent synthesis. In both cases, the yield of macro-RAFT agent exceeds 90%.

Block copolymers have been synthesized as follows: Macro-RAFT agent was dissolved in the mixture of VIM and glacial acetic acid taken in the molar ratio as 1:5. Then, AIBN was added to the final mixture. Other operations are similar to described above, except polymerization was conducted at 70 °C for 72 h. After polymerization, the reaction mixture was cooled in liquid nitrogen, then diluted with water, subjected to dialysis against water using dialysis sack with cut-off 3 kDa for 3 days and lyophilized. The total monomer conversion was determined by gravimetry considering the weight of macro-RAFT agent in the probe. Table 4 summarizes the formulations of reaction mixtures for block copolymer synthesis. In both cases, the yield of macro-RAFT agent exceeds 90%.

### 3.2. Instrumentation

The average molecular weights and dispersity (Ð = M_w_/M_n_) were determined by the size exclusion chromatography (SEC). The SEC measurements were performed in DMF containing 0.1 wt% of LiBr at 50 °C with a flow rate of 1.0 mL/min using a chromatograph GPC-120 “PolymerLabs” equipped with refractive index and with two columns PLgel 5 µm MIXED B for MW range 5 × 10^2^–1 × 10^7^. The SEC system was calibrated using narrow dispersed linear poly(methyl methacrylate) standards with MW ranging from 800 to 2 × 10^6^ g mol^−1^. A second-order polynomial was used to fit the log_10_*M* versus retention time dependence. All copolymers were subjected to methylation by diazomethane before analysis.

The average hydrodynamic radius R_h_ of the particles of polymeric dispersions was determined by dynamic light scattering (DLS). DLS measurements were performed by a static/dynamic compact goniometer (DLS/SLS-5000, ALV, Langen, Germany). A HeNe laser with a power of 22 mW emitting a polarized light at λ = 633 nm was used as the incident beam. The studies were conducted at 23 ° C, and a scattering angle of 90°. At each temperature, the samples were left for 30 min to equilibrate, at chosen temperatures above the polymer cloud point, the samples were also left for 2 h to study the stability of the solution. Typical weight concentrations of the samples were 0.01 and 0.05 g/L; bidistilled water and 1,4-dioxane were used as solvents. The pH of the solution was corrected by the addition of small amounts of 1M KCl and 1M NaOH. Distributions over decay times were obtained using a nonlinear regularized inverse Laplace transformation method (CONTIN) [63].

The copolymer composition was analyzed by ^1^H nuclear magnetic resonance (NMR) spectroscopy using spectrometer “VARIAN XR-400” at 400 MHz. The samples were prepared by dissolving the copolymers in DMSO-d_6,_ which was used as an internal standard.

Fluorescence spectra were recorded using F-4000 spectrofluorometer (Hitachi, Japan) at excitation wavelength λ_ex_ = 340 nm. The samples were prepared in acetate buffer with pH 4.5 and Tris buffer with pH 9. After 1 h of incubation at temperature of 25 °C or 55 °C the samples were analyzed in thermostatic cell of the spectrofluorometer.

## 4. Conclusions

To summarize, in the present research, we have performed the first comparative study of the effects of both temperature and pH on the conformational behavior of diblock and triblock copolymers of PNIPA and PVIM. Focusing on this, we have synthesized PNIPA_28_-*b*-PVIM_62_-*b*-PNIPA_29_ and PNIPA_60_-*b*-PVIM_90_ block copolymers using RAFT polymerization mediated by symmetric and asymmetric RAFT agents. This led to the different structure of block copolymers including the number and the sequence of the blocks, the locus of trithiocarbonate group, hydrophobicity of the chain-end groups. All these factors affect the properties of the block copolymers in aqueous solution at different temperatures and pH.

First, temperature responsive properties of both PNIPA-1 and PNIPA-2 are different due to the locus of trithiocarbonate group and various nature of the chain-end groups. These variations reveal in the different values of R_h_ and temperature range corresponding to the phase transition, while clouding point retains equal to ~32° C.

The incorporation of PVIM block shifts the temperature range of the phase transition and gives pH-sensitivity to the block copolymers. Cloud points of PNIPA_60_-*b*-PVIM_90_ are ~43 °C and ~ 37 °C at pH 3 and 9, respectively; and for PNIPA_28_-*b*-PVIM_62_-*b*-PNIPA_29_ they are ~35 °C and 31 °C at pH 3 and 9. At both acidic and alkali conditions, triblock copolymer containing internal PVIM block with P_n_ close to total P_n_ of both PNIPA blocks is characterized by lower clouding points and R_h_ values as compared to diblock copolymer with P_n_ of PVIM block one-half higher than that of PNIPA block. At 25° C, i.e., below cloud point, the R_h_ value of the block copolymer particles at pH 3 is lower than at pH 9 independently of the number and the length of the blocks. Hence, hydrophobization of PVIM block may be responsible for self-assembling of block copolymer at high pH. Around cloud point temperature, independently of pH, the *R_h_* value for triblock copolymer rises sharply, achieves the maximum value, then falls and reaches the constant value, while for diblock copolymer, it steadily grows after reaching cloud point. The information about polarity of microenvironment around polymer obtained by fluorescence method using pyrene probe accords with DLS data.

Thus, the locus of PVIM block may play an important role in the stimuli-responsive properties of block copolymers, expanding the area of PNIPA-PVIM application in the advanced materials design. The double stimuli-responsive cationic block-copolymers are prospective components for antimicrobial formulations [64], nanocarriers [65], gene-delivery vehicles [66], and surfactants [67].

## Figures and Tables

**Table 1:** Molecular characteristics of the synthesized block copolymers.

Sample	macroRAFT	M_n_(SEC), kg⋅mol^−1^	Đ	*F*_VIM_, mol.%	P_n_ (VIM)	M_n_(NMR), kg⋅mol^−1^
Block-1	PNIPA-1; P_n_ = 57	10.2	1.19	52	62	12.3
Block-2	PNIPA-2; P_n_ = 60	12.6	1.12	60	90	15.2

**Table 2:** Cloud point temperatures T_cp_ and hydrodynamic radii R_h_ of obtained thermoresponsive polymers.

Sample	pH	T_cp,_ C°	ΔT_cp,_ C°	R_h_, nm below T_cp_	R_h_, nm above T_cp_
PNIPA-1	3.0–9.0	32.6 ± 0.2	7.1	1 ± 0.1	-
PNIPA_28_-*b*-PVIM_62_-*b*-PNIPA_29_	3.0	35.1 ± 0.3	15.6	2 ± 0.3	377 ± 10
	9.0	31.2 ± 0.2	6.8	4 ± 0.3	736 ± 16 *
PNIPA-2	3.0–9.0	32.7 ± 0.2	11.6	8 ± 1	-
PNIPA_60_-*b*-PVIM_90_	3.0	43.0 ± 0.4	10.2	3 ± 0.3	47 ± 6
	9.0	37.5 ± 0.3	7.5	5 ± 0.5	543 ± 11 *

* The sample precipitates with time when heated a few degrees above the T_cp_.

**Table 3:** Formulations of reaction mixtures for macro-RAFT agent synthesis.

Macro-RAFT Agent	NIPA, g	1,4-Dioxane, mL	RAFT Agent	AIBN
PNIPA-1	2	2	BTC, 0.07 mol/L (0.0812 g)	10^−3^ mol/L 0.07 mL solution in 1,4-dioxane (0.009 g in 1 mL)
PNIPA-2	1	1	CPDTC, 0.07 mol/L (0.048 g)	10^−3^ mol/L 0.1 mL solution in 1,4-dioxane (0.0026 g in 0.8 mL)

**Table 4:** Formulations of reaction mixtures for block copolymer synthesis.

Macro-RAFT Agent	VIM, mL	CH_3_COOH, mL	AIBN, g	Conversion, %
PNIPA-1; 0.23 g	1.2	3.8	0.002	12.8
PNIPA-2; 0.20 g	1.2	3.8	0.002	24.1

## Data Availability

Not applicable.

## Supplementary Materials

The supporting information can be downloaded at: https://www.mdpi.com/article/10.3390/ijms24010879/s1.
