# Supplementary material for: Double Stimuli-Responsive di- and Triblock Copolymers of Poly(N-isopropylacrylamide) and Poly(1-vinylimidazole): Synthesis and Self-Assembly
Source: Int J Mol Sci. 2023 Jan 3;24(1):879. doi: 10.3390/ijms24010879 (PMC9820948; doi:10.3390/ijms24010879)
Supplement: Supplementary file 1 [file ijms-24-00879-s001.zip › ijms-2125946-supplementary.pdf]

Supplementary Information

Double stimuli-responsive di- and triblock copolymers of poly(N-isopropyl acrylamide) and poly(1-vinyl imidazole): synthesis and self-assembly

Elena Yu. Kozhunova [1,2,*], Anna V. Plutalova [2], Andrey V. Sybatchin [2], Alexander V. Chertovich [3,1] and Elena V. Chernikova [2,*]

1 Lomonosov Moscow State University, Faculty of Physics, Russian Federation, 119991, Moscow, Lenin Hills, 1, bld. 2
2 Lomonosov Moscow State University, Faculty of Chemistry, Russian Federation, 119991, Moscow, Lenin Hills, 1, bld. 3
3 Semenov Federal Research Center for Chemical Physics, Moscow, 119991, Russian Federation, 119991, Moscow, Kosygina str., 4
* Correspondence: kozhunova@polly.phys.msu.ru (EK), chernikova_elena@mail.ru (EC)

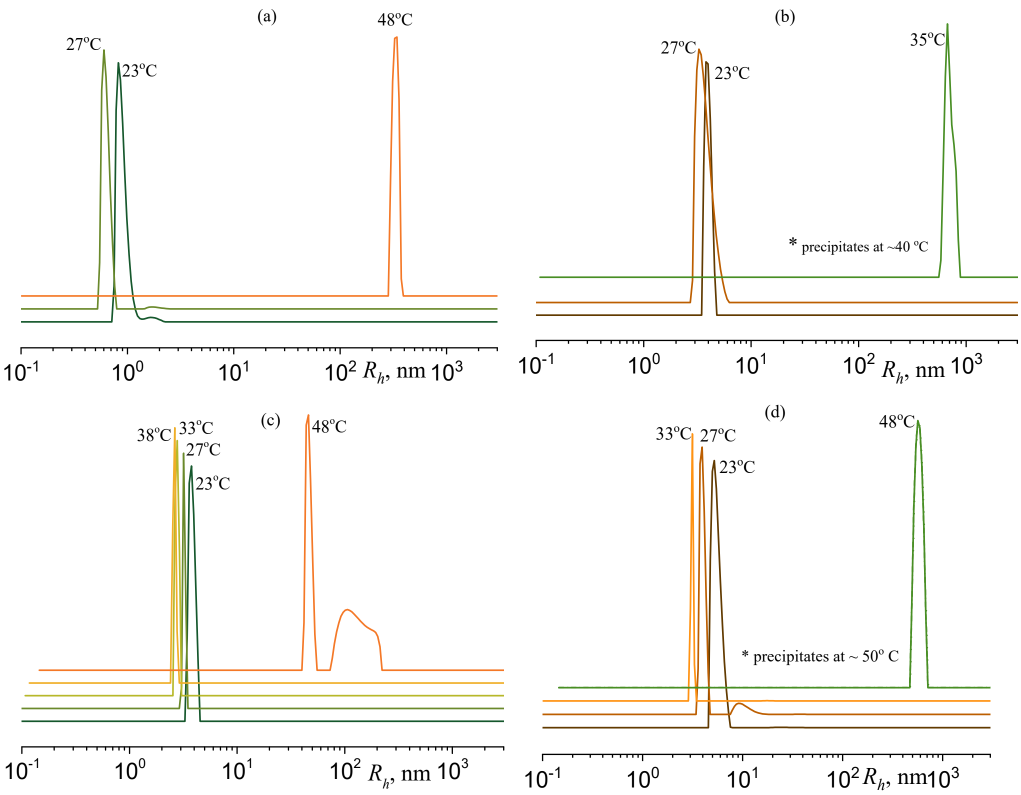

Figure S1. The hydrodynamic radius R_h distribution curves at the selected temperatures for PNIPA$_{28}$-*b*-PVIM$_{62}$-*b*-PNIPA$_{29}$ at pH 3 (a) and pH 9 (b); PNIPA$_{60}$-*b*-PVIM$_{90}$ at pH 3 (c) and pH 9 (d). The measurements were taken under the scattering angle of 90°, R_h is number-averaged.

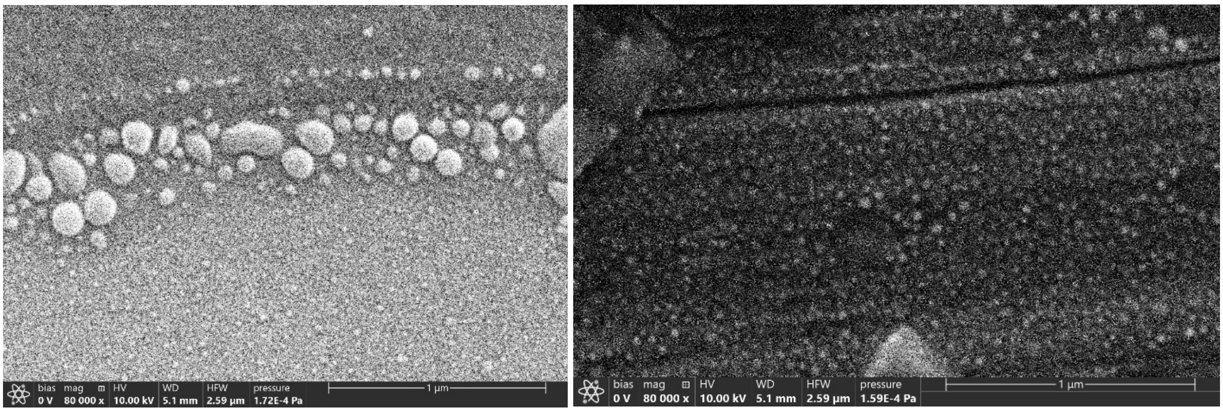

Figure S2. Scanning electron microscopy images of the dried polymer solutions of for $PNIPA_{28}$-*b*-$PVIM_{62}$-*b*-$PNIPA_{29}$ at pH 3 (a) and $PNIPA_{60}$-*b*-$PVIM_{90}$ at pH 3 (b).

Scanning electron microscopy (SEM) images were obtained using a Prisma E microscope (Thermo Scientific, Brno, Czech) with 10 kV accelerating voltage in the high vacuum mode. The sample preparation procedure was the following. The small drops of 0.2 mg\ml of polymer solution were heated to 60°C, placed on heated silica wafers and left to dry for 24 hours. Directly before the SEM measurements, the samples were coated with 10 nm layer of gold by Q150R ES plus sputter coater (Quorum Technologies, East Sussex, UK). Scanning electron microscopy was performed in the ACBS Center of the Collective Equipment (no. 506694, FRCCP RAS) by Dr. Alexander Gulin.
